# Generalized Pustular Psoriasis: Current Treatment and Innovative Therapies

**DOI:** 10.7759/cureus.103366

**Published:** 2026-02-10

**Authors:** José Dario Martínez Villarreal, Mariano Garcia-Campa, Manuel Hernandez-Fuentes, Jesús Alberto Cárdenas-de la Garza, Antonio Martorell

**Affiliations:** 1 Internal Medicine and Dermatology, Christus Muguerza Alta Especialidad, Monterrey, MEX; 2 Internal Medicine, Christus Muguerza Alta Especialidad, Monterrey, MEX; 3 School of Health Sciences, Universidad de Guadalajara, Guadalajara, MEX; 4 Dermatology and Rheumatology Service, University Hospital “Jose E. González”, Universidad Autónoma de Nuevo León, Monterrey, MEX; 5 Dermatology and Venereology Service, Hospital of Manises, Valencia, ESP

**Keywords:** acute generalized pustular psoriasis, autoimmune flare up, generalized pustular psoriasis (gpp), psoriasis and autoimmunity, spesolimab

## Abstract

Background: Generalized pustular psoriasis (GPP) is a rare, life-threatening auto-inflammatory disease driven by IL-36 pathway dysregulation leading to recurrent flares of widespread erythema, sterile pustules, and systemic symptoms. Real-world data on spesolimab - the first U.S. Food and Drug Administration (FDA)/European Medicines Agency (EMA)-approved GPP therapy - remains scarce, particularly outside Europe.

Methods: We performed a retrospective case series with prospective follow-up (2010-2025) following Consensus-based Clinical Case Reporting (CARE) guidelines. Nine patients diagnosed according to European Rare and Severe Psoriasis Expert Network (ERASPEN) criteria by board-certified dermatologists were included. Data on demographics, triggers, clinical features, complications, and treatments were collected from electronic medical records.

Results: Nine patients (100%) aged 3-61 years, including five (55.6%) female patients, were included. Obesity was the most common comorbidity (n=6, 66.7%). Steroid withdrawal was the most common trigger (n=5, 55.6%). All patients presented with generalized erythema, sterile pustules, and pain; complications observed were acute respiratory distress syndrome (ARDS) in two patients (22.2%) and acute cholangitis in one patient (11.1%). Six flares required inpatient care. Spesolimab achieved 100% flare-free status at 12 months versus 50-75% recurrence with non-IL-36 agents. A tiered treatment algorithm was proposed, prioritizing rapid IL-36 blockade in severe flares.

Conclusion: This largest Latin American GPP cohort reveals an earlier onset in pediatric patients and high complication rates. Spesolimab shows superior flare control, but access barriers persist. Standardized protocols and genetic studies in diverse populations are urgently needed.

## Introduction

Generalized pustular psoriasis (GPP) is a severe, systemic auto-inflammatory disorder distinct from plaque psoriasis vulgaris (PV). Affecting ~1% of psoriasis patients, GPP features recurrent flares of widespread sterile pustules on erythematous skin, often with systemic manifestations such as fever, leukocytosis, and life-threatening complications like acute respiratory distress syndrome (ARDS), cholangitis, and sepsis [1-3]. Unlike PV’s adaptive IL-23/IL-17 axis, GPP is driven by innate immune dysregulation via hyperactive IL-36 signaling in keratinocytes and neutrophils [4]. Loss-of-function IL-36RN mutations (encoding IL-36 receptor antagonist) are found in 20-60% of cases, with CARD14 gain-of-function variants also implicated [5]. Despite the U.S. Food and Drug Administration (FDA)/European Medicines Agency (EMA) approval of spesolimab (anti-IL-36R monoclonal antibody) in 2022, no international treatment consensus exists [6]. The Effisayil-1 trial demonstrated better pustule clearance with spesolimab as early as one week after initiation compared with placebo, but real-world integration with conventional agents - especially in non-European populations - remains underexplored [7-10]. Latin American data are virtually absent, and Mexican prevalence and genetic profiles are unknown. In this study, we present the largest GPP case series from Latin America, with the objective of evidencing real-world spesolimab outcomes and a pragmatic management algorithm for variable-resource settings.

## Materials and methods

This study adheres to the Consensus-based Clinical Case Reporting (CARE) guidelines [11]. All participants provided written informed consent prior to inclusion in this case series. The informed consent process was conducted in accordance with the ethical principles outlined in the Declaration of Helsinki (1975, revised 1983) and fulfilled the requirements for good clinical practice and patient autonomy [11]. Patients (or their legal guardians when applicable) received comprehensive, age-appropriate, and language-congruent information regarding the study’s purpose, procedures, retrospective data collection, prospective follow-up, voluntary nature of participation, right to withdraw at any time without consequence to their medical care, and measures implemented to ensure confidentiality. Written consent was obtained and documented in each patient’s medical record before any study-related data were accessed or recorded. No participant was included without documented evidence of adequately obtained, voluntary informed consent. 

Patient selection

We conducted a multicenter case series on patients diagnosed with GPP. Patients were recruited from dermatology inpatient and outpatient clinics in Monterrey, Mexico (n=8, 88.9%), and Valencia, Spain (n=1, 11.1%), between January 2010 and December 2024. Inclusion criteria were patients presenting with their first documented GPP flare diagnosed based on clinical history, cutaneous manifestations such as widespread erythema with primary sterile pustules, associated systemic symptoms, and fulfillment of the European Rare and Severe Psoriasis Expert Network (ERASPEN) consensus criteria [12]. Diagnosis was confirmed by a board-certified dermatologist. Exclusion criteria included alternative diagnoses such as acute generalized exanthematous pustulosis (AGEP), sub-corneal pustular dermatosis, pustular drug eruptions, or pustulation confined to pre-existing psoriatic plaques. Patients were consecutively included to minimize selection bias. Enrolled patients had at least one year of follow-up data available.

Data collection 

Data were collected retrospectively from electronic medical records for the period January 2010 to December 2024, with prospective follow-up extending until July 2025. Variables included baseline demographic and disease characteristics, therapies, and follow-up. Patients were followed for at least one year in consultation with their dermatologist, collecting flare recurrence and characteristics prospectively. All personal identifiable information was de-identified and encrypted to protect patient privacy.

Statistical analysis

Descriptive statistics were used to summarize the data. Categorical variables were reported as frequencies and percentages. No inferential statistics were performed due to the nature of the study. Statistical analyses were performed using IBM SPSS Statistics v25 (IBM Corp., Armonk, USA).

## Results

A total of nine patients (100%) were included in this case series. The age range at presentation was 3-63 years, with most patients being female (n=5, 55.6%). Eight (88.9%) Hispanic patients and one (11.1%) White patient were included. Obesity, present in six patients (66.7%), was the most frequent comorbidity. Similarly, six (66.7%) patients had plaque psoriasis. The most common trigger - systemic steroids withdrawal - was present in five patients (55.6%), followed by infection in one patient (11.1%) and anti-IL-23 in one patient (11.1%) (Table 1).

**Table 1:** Patient baseline characteristics and therapeutic approach ARDS: acute respiratory distress syndrome; HTN: hypertension; GPP: generalized pustular psoriasis; GPPASI: Generalized Pustular Psoriasis Area and Severity Index

Case no.	Gender	Age (years)	Trigger	Comorbidity	Previous psoriasis	Clinical presentation	Outpatient / inpatient	Initial treatment	Complications	Six-month follow-up	Maintenance	12-month follow-up
1	Male	61	Systemic steroids	Obesity	No	Generalized erythema + multiple pustules + pain; GPPASI: 7.4	Outpatient	Cyclosporine	None	No flare; GPPASI: 3.0	None	No flare; GPPASI: 0.4
2	Female	31	Systemic steroids	None	No	Generalized erythema + multiple pustules + pain; GPPASI: 4.3	Outpatient Inpatient	Cyclosporine	None	Flare; GPPASI: 6.3	Risankizumab	No flare; GPPASI 1.0
3	Female	24	Infection	Down syndrome, obesity	Plaque psoriasis	Generalized erythema + multiple pustules + pain; GPPASI: 7.7	Inpatient	Infliximab	ARDS	No flare; GPPASI 2.1	Adalimumab	No flare; GPPASI 0.9
4	Male	42	Systemic steroids	HTN, Obesity	Plaque psoriasis	Generalized erythema + multiple pustules + pain; GPPASI: 8.9	Inpatient	Etanercept	ARDS	No flare; GPPASI: 2.6	Adalimumab	No flare; GPPASI: 0.9
5	Female	30	Systemic steroids	Obesity	Plaque psoriasis	Generalized erythema + multiple pustules + pain; GPPASI: 7.4	Inpatient	Infliximab	Cholangitis	Flare; GPPASI: 6.8	Risankizumab	No flare; GPPASI: 0.9
6	Female	3	Systemic steroids	Infection	No	Annular plaques + erythema + pain; GPPASI: 3.0	Inpatient	Cyclosporine	None	No flare; GPPASI: 1.2	None	No flare; GPPASI: 0.6
7	Female	38	None	Obesity	Plaque psoriasis	Multiple plaques + erythema + pustules + pain; GPPASI: 6.1	Outpatient	Adalimumab	None	No flare; GPPASI: 1.9	Adalimumab	Moderate flare; GPPASI: 0.7
8	Male	63	None	None	Plaque psoriasis, two GGP flares	Generalized erythematosquamosus plaques + pustules + pain; GPPASI: 2.8	Outpatient	Spesolimab: two doses	None	No flare; GPPASI: 1.6	None	No flare; GPPASI: 0.8
9	Male	45	Anti-IL-23	Obesity	Plaque psoriasis	Multiple plaques + erythema + pustules + pain; GPPASI: 4.1	Inpatient	Spesolimab: two doses	None	No flare; GPPASI: 1.1	Secukinumab	No flare; GPPASI: 0.4

**Figure 1:**
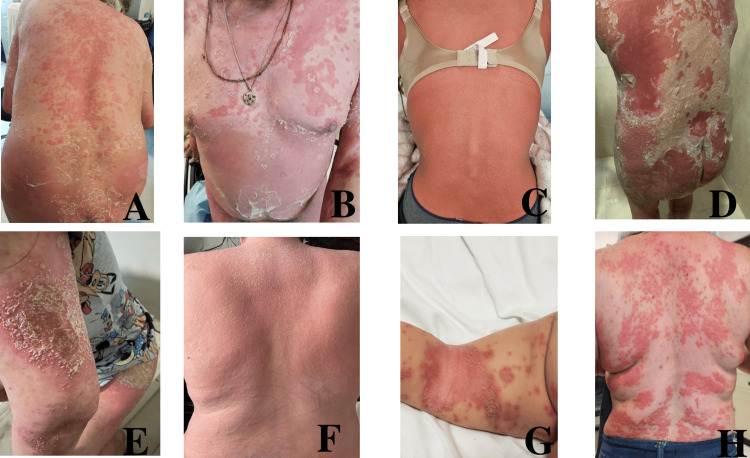
Clinical manifestations of GPP A-B: Multiple plaques with erythema and confluent small sterile pustules and areas of desquamation (Case 1, initial presentation). C: Red erythema with multiple small pustules and a burning sensation on the back of this female patient (Case 2, flare). D: Deep erythema with confluence of pustules, so called “lake of pus”, and a sensation of burning pain on the back of this female patient (Case 5, initial presentation). E: Plaque with multiple pustules over erythema in the arm of this female patient (Case 5, initial presentation); F: Back with erythema and multiple small pustules, and sensation of burning over the back of this female patient (Case 5, flare); G: Annular plaques with erythema and areas with small pustules in this girl child (Case 6, initial presentation); H: Multiple plaques with erythema and small papules and sterile pustules (Case 7, initial presentation) GPP: generalized pustular psoriasis

At presentation, all patients presented with a chief complaint of generalized burning sensation, pain, erythema, and multiple sterile pustules. Four (44.4%) patients presented with plaques, and one pediatric patient presented with annular plaques. Eight (88.9%) patients presented with lesions in ≥30% of their body surface area (BSA), predominantly in the dorsal region, abdomen, and upper extremities, as shown in Figure 1. The decision to manage two patients (22.2%) in the outpatient setting was made due to the SARS-CoV-2 pandemic and, in one patient (11.1%), due to a moderate GPP flare.

Treatment modalities, maintenance, and follow-up are presented in Table 1. Spesolimab showed no flares with almost clear cutaneous lesions after 7-20 days. None of the patients presented with systemic complications due to GPP. One (33.3%) of the three patients treated with cyclosporine presented with a flare after previous remission was achieved. This patient presented with less intense manifestations at the initial presentation and was treated again with cyclosporine plus risankizumab as maintenance therapy with no flares at one-year follow-up. Patients managed with infliximab presented with systemic complications due to GPP; one presented with ARDS managed in the intensive care unit and acute cholangitis. The patient managed with etanercept for the initial presentation was also complicated by ARDS. After remission was achieved, the patient was treated with adalimumab as maintenance therapy with no flares at follow-up. When adalimumab was used as maintenance after infliximab or etanercept, no flares were observed.

**Figure 2:**
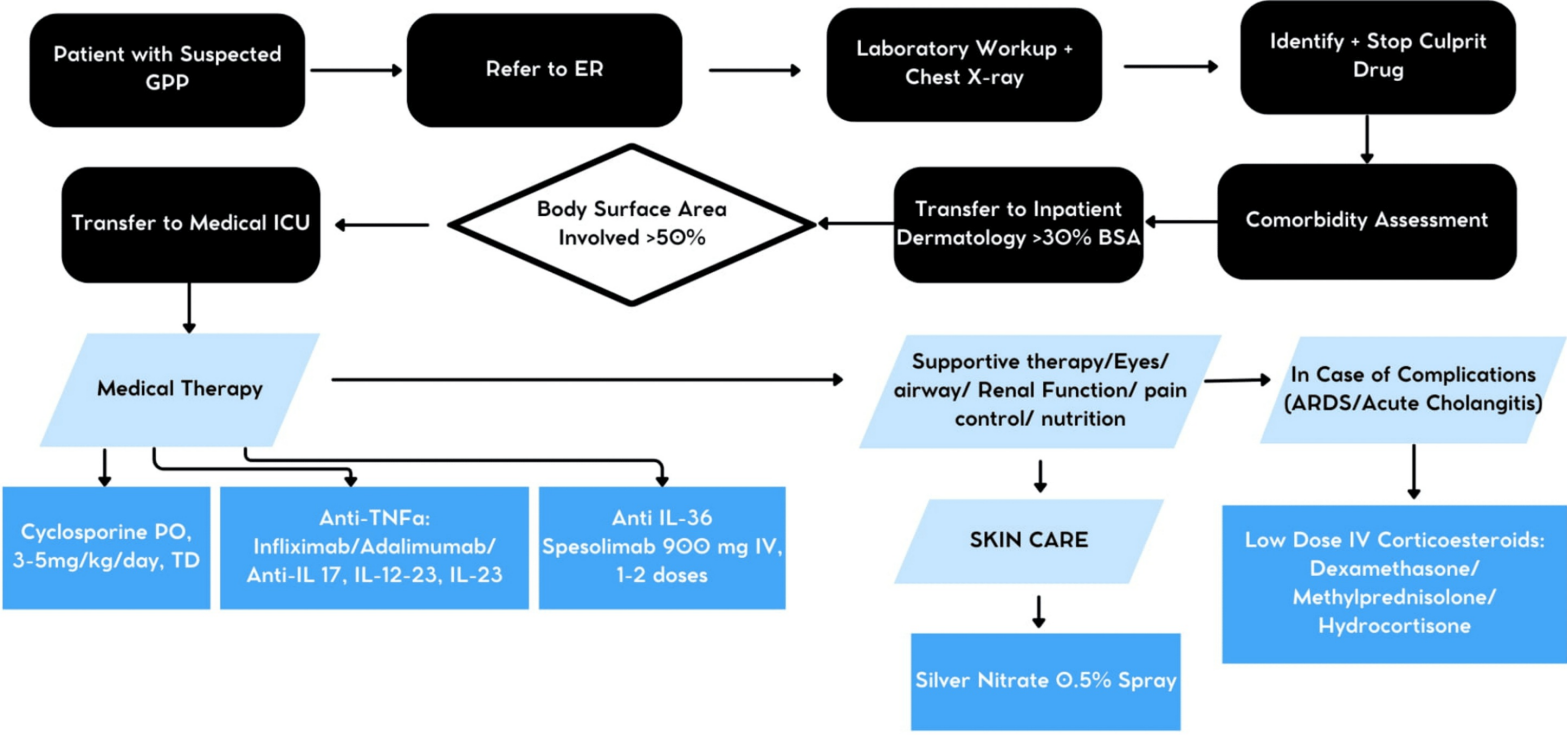
GPP approach and treatment algorithm ARDS: acute respiratory distress syndrome; BSA: body surface area; ER: emergency room; ICU: intensive care unit; PO: per os; TD; twice daily; GPP: generalized pustular psoriasis The figure was designed and developed by the authors.

Skin lesions were present in all patients. We managed pustules and plaques with silver nitrate spray to support healing and prevent infections. This strategy, combined with individualized analgesics, promoted early resolution and less pain. Silver nitrate spray resulted in drier lesions, less scaling, and peeling. Erythema was mainly treated with systemic therapy, as shown in Figure 2.

## Discussion

Our investigation revealed that spesolimab prevents flares in GPP, achieving flare-free status in 12 months. Obesity was the most associated condition (n=6, 66.7%), while discontinuation of systemic corticosteroids was the primary precipitating factor (n=5, 55.6%). These results emphasize the value of targeting the IL-36 pathway for controlling acute GPP episodes, though access barriers to advanced biologics pose difficulties in low-resource environments. Severe systemic inflammation was the main driver of complications, with ARDS and acute cholangitis confined to hospitalized patients experiencing intense flares. The three-year-old child in our series exhibited annular pustular lesions, an uncommon presentation in adult cases, which may point to age-related variations in IL-36 signaling.

Prior European studies have demonstrated superior flare resolution with IL-36 inhibition in GPP, aligning with outcomes from a Mexican patient [13-15]. Such evidence calls for efforts to address global disparities in treatment availability. We suggest a practical management flowchart, as shown in Figure 2, suited for limited-resource settings, relying on clinical evaluation and routine lab tests for feasibility and reproducibility [16-18]. This approach can be paired with low-cost measures focused on avoiding known triggers. Determining specific biomarkers or clinical indicators that forecast response to IL-36 inhibitors could pave the way for more structured treatment guidelines, potentially reducing reliance on trial-and-error escalation [19]. These could assist in decisions regarding hospitalization, choice of biologics, and early detection of complications - particularly relevant here as ARDS and cholangitis arose in critical inpatient flares [20,21]. Management must always prioritize patient preferences, including their grasp of GPP's chronic relapsing course and views on sustained treatment. This alignment ensures that therapeutic strategies reflect patients’ priorities. In GPP care, patients often value sustained remission and improved daily functioning over intensive immunosuppression risks. Dermatologists should proactively engage in treatment dialogues during acute episodes to foster patient involvement in ongoing care [22]. Starting biologics offers a key moment to review risks, benefits, and outlook using validated scores such as the Generalized Pustular Psoriasis Area and Severity Index (GPPASI) and the Generalized Pustular Psoriasis Physician Global Assessment (GPPPGA), matching interventions to personal values and preferences [23-28]. Challenges may include balancing treatment potency with availability; denying access to spesolimab purely on financial grounds raises ethical concerns. Besides, evidence is limited to the Effisayil trial, in which infections and systemic drug reactions were also observed [8,29,30]. Healthcare providers must highlight the drawbacks of therapeutic agents and periodically follow up on clinicians' and patients' goals. Socioeconomic factors should inform but not dictate choices; optimal evidence-based care remains the standard. Integrating patient-centered elements with tools like our flowchart may prove especially beneficial in limited-resource areas, where collaborative decisions can mitigate inequalities and ease overall disease burden.

In low-access contexts, merging severity assessment, trigger profiles, and biologic availability can dictate sequential therapy recommendations and show reliable prognostic value. Educating patients enhances informed decision-making. Proactive flare contingency planning optimizes efficiency, channeling support to key areas like weight control and infection prevention. Modifying long-term regimens might boost compliance but demands vigilance for undetected inflammation. Larger initiatives should aim at expanding biologic distribution in low-resource settings and strengthening dermatology services for trigger management. Further, future studies must incorporate patient perspectives and flexible instruments to promote equitable involvement [31]. 

This work examines a Latin American cohort, evidencing a management flowchart [32]. Limitations of our study encompass the modest cohort size inherent to GPP's infrequency and absence of molecular genotyping, constraining broader applicability. Results were obtained from observational data with a small sample size of patients; thus, specific efficacy is difficult to estimate. Nonetheless, the data provide orientation regarding the efficacy of IL-36 inhibition and advocate for customized strategies in GPP treatment. Multicentric studies to provide evidence-based cut-off values for our algorithm could improve specific decision-making.

In our real-world study, two patients treated with spesolimab had no flares at follow-up, and their skin manifestations were resolved in 1-2 weeks. None of them presented with systemic complications due to GPP. In contrast, the patients who were managed with infliximab presented with systemic complications due to GPP. One patient who was managed with etanercept was complicated by ARDS but had no flares at follow-up and was given adalimumab as maintenance therapy. When adalimumab was used as maintenance after infliximab or etanercept, no flares were observed. Adverse events after the use of anti-TNFα biologics can result in annular GPP-like lesions. Similarly, studies show that infections occur in almost in 50% of patients receiving spesolimab [8,29,30]. Fast-acting drugs such as cyclosporine and infliximab are available in different regions; however, spesolimab has limited accessibility.

The proposed GPP algorithm covers the initial approach and management of patients presenting in the outpatient setting or the emergency department. Generalized or disseminated sterile pustules associated with painful erythema should raise clinicians’ suspicion. Initial routine laboratory workup and chest radiography should guide clinicians to determine which patient has a high risk or may present with GPP complications. Clinical history is also crucial to identify GPP triggers. If physical examination reveals skin disease covering >30% of their BSA, it can be considered a criterion for in-hospital management, whereas patients with lesions in >50% of their BSA can be transferred to the intensive care unit. In-hospital management should not be delayed. Spesolimab in one or two doses has shown promising results [8,29,30]. Severe presentations may include skin lesions and systemic manifestations. In our study, systemic manifestations were managed with low-dose systemic steroids to reduce inflammation. We managed pustules and plaques with silver nitrate spray to support drying and prevent wound infections. This strategy, combined with individualized analgesics, promoted early resolution with less pain. Looking ahead, glucagon-like peptide-1 receptor agonists (GLP-1RA) may soon gain prominence as adjuvant therapy for managing obesity, a frequent comorbidity in this patient population [33].

## Conclusions

This case series reinforces GPP’s distinct pathogenic profile, emphasizing diagnosis and individualized management. The integration of novel therapies is challenged by accessibility. Our proposed treatment algorithm provides a practical framework for clinicians, advocating for timely intervention. Evidence-based thresholds would integrate decision-making in clinical practice. 
